# The effect of student-led healthcare career experience activity on nursing students’ professional commitment: Study protocol for a randomized cross-over trial

**DOI:** 10.1371/journal.pone.0340372

**Published:** 2026-06-16

**Authors:** Shilai Yang, Yanli Lv, Biaojun Yu, Weiling Chen, Jin Peng, Mohd Afifuddin Mohamad

**Affiliations:** 1 Department of Community Health, Pusat Kanser Tun Abdullah Ahmad Badawi, Universiti Sains Malaysia, Penang, Malaysia; 2 School of Nursing, Quanzhou Medical College, Quanzhou, Fujian, China; Public Library of Science, UNITED KINGDOM OF GREAT BRITAIN AND NORTHERN IRELAND

## Abstract

**Background:**

Career experience is a valuable approach to stimulate students’ learning interest and reshape their understanding of career pathways. However, the interaction effects between implementers and experiencers, particularly the benefits of the implementers, remain poorly understood. Furthermore, the role of peer-assisted learning in enhancing the effectiveness of career experience activities has not been thoroughly explored. This study aims to evaluate the impact of student-led healthcare career experience activity on nursing students’ professional commitment, academic self-efficacy and learning burnout.

**Methods:**

This is the study protocol for an open-label, randomized stepped-wedge crossover trial. This study will be set in a medical vocational college in China and will include 110 nursing students, who will be randomly allocated (1:1:1) to one of three groups. In a sequential, stepped-wedge manner, the groups will cross over from control conditions (Waitlist and Observer) to the intervention, where they will lead a healthcare career experience activity. The primary outcome of professional commitment, as well as the secondary outcomes of academic self-efficacy and learning burnout, will be measured via self-reported questionnaires at baseline and three subsequent time points (after the 4th, 8th, and 12th activity sessions). Data will be analyzed using Generalized Estimating Equation (GEE) analysis.

**Conclusion:**

This protocol details a student-led healthcare career experience activity, grounded in Kolb’s Experiential Learning Theory and Life Cycle Theory, and designed to enhance nursing students’ professional commitment. This trial is expected to provide empirical evidence on the feasibility of this peer-assisted learning model applied in a student-led nursing educational scenario, which facilitates both peer-to-peer and near-peer interactions. Furthermore, the outcomes are expected to inform the future development of student-led educational strategies.

**Trial registration:**

This study is registered in Chinese Clinical Trial Registry (ChiCTR2300070880). https://www.chictr.org.cn/bin/project/edit?pid=195337

## 1. Introduction

In recent years, global nurse turnover rates have risen, particularly among newly graduated nurses [1]. This trend presents a key challenge to the stability of the nursing workforce [2]. Factors such as transition shock, burnout, and emotional exhaustion contribute to this issue, which was amplified after the COVID-19 pandemic [3,4]. As a result, controlling turnover has become a critical priority for nursing educators and managers. Studies confirm that professional commitment is a core predictor of turnover intention. Strengthening this commitment through early interventions during school is an effective strategy to reduce future turnover [5,6]. According to Career Construction Theory, career experience is an essential part of career development [7]. It serves as a form of social support and plays a vital role in enhancing students’ professional commitment [8,9].

As a dynamic and interactive process, career experience has been shown to spark learning interest, refine occupational perceptions, and enhance planning skills [10–15], However, traditional career experience models face two major challenges in practice. The first is a high dependency on resources. Traditional models often require qualified instructors and real clinical or campus venues [16–18]. This places significant pressure on institutional finances and human resources, making such activities difficult to sustain [19]. The second problem is the limited perspective of existing research. Previous studies have focused almost exclusively on the benefits for experiencers, while largely ignoring the effects on the activity implementers, such as medical staff or teachers [20,21]. This represents a significant research gap. To address these challenges, the peer-assisted learning (PAL) model offers a potential solution.

The core of PAL is that students acquire knowledge and skills through the active support of their peers [22]. The advantage of this model is transforming resource limitations into a teaching opportunity. By allowing students to act as facilitators, this approach greatly reduces the reliance on professional faculty. More importantly, it shifts students from being passive recipients of knowledge to active creators of it. In the process of teaching others, they can develop their leadership, communication, and organizational skills [23–25]. These are key competencies for their future careers that are difficult to achieve in traditional teacher-led models. Moreover, peer interaction can create a more equal learning environment [22]. This may help to increase student motivation and potentially reduce the learning burnout prevalent in nursing programs [26,27]. Previous studies show that student-led activities can effectively enhance professional identity [28–30]. Despite these advantages, the PAL model also faces its own challenges, such as a potential lack of student motivation or conflict within groups, as well as the need for structured training and resource support. All these factors must be considered in the design [31].

Therefore, this study aims to address the following research questions: (1) How was the student-led healthcare career experience activity developed? (2) How will the key outcomes (professional commitment, academic self-efficacy, and learning burnout) change across four measurement time points (T0-T3)? (3) What are the differences in outcomes between Group 1 at T1 and T2? (4) What are the differences in outcomes among all groups at T3?

## 2. Theoretical and conceptual model

The theoretical foundation for this study is Kolb’s Experiential Learning Theory [32]. It emphasizes that learners construct knowledge through the active transformation of experience. Experiential learning has been widely applied in nursing education to facilitate active learning and develop practical skills [33,34]. However, many of these applications remain teacher-led, focusing on students as passive recipients of pre-structured experiences. The potential for students to learn by actively creating and facilitating an experience for others, particularly in relation to their own professional identity and learning attitudes, remains underexplored.

To structure the content of the intervention, the Life Cycle Theory was utilized. Nursing roles were categorized according to three major life stages: juvenile, young and middle-aged, and elderly. This framework provides a comprehensive and relatable structure for demonstrating the critical role of nursing across a person’s entire lifespan.

**Fig 1:**
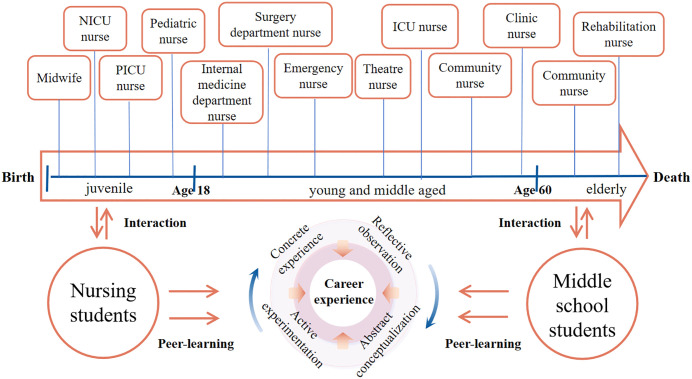
Conceptual framework of healthcare career experience activity.

These two theories were integrated to create the student-led activity. The Life Cycle Theory provided the structured content of the nursing stations. Kolb’s Experiential Learning Theory provided the process through which the nursing students, as implementers, would learn. For each station, the nursing students will guide experiencers through the four stages of experiential learning. Nursing students themselves will actively engage in a learning cycle by gaining concrete experience by leading the activities, observing experiencers’ reactions and reflecting on their teaching performance, and subsequently conceptualizing these insights to refine their understanding of their professional role. This process is designed to deepen their own understanding of nursing roles and enhance their professional commitment. As a secondary benefit, this activity also helps high school students understand the significance of nursing across the human life cycle (Fig 1).

## 3. Methods

### 3.1. Study design

**Fig 2:**
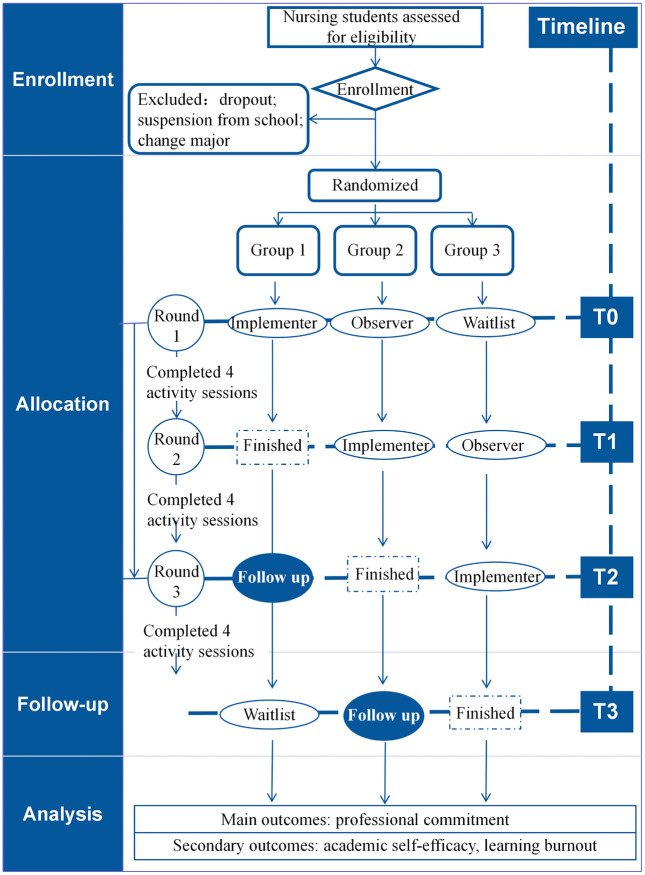
The flowchart of student enrollment and group allocation.

**Fig 3:**
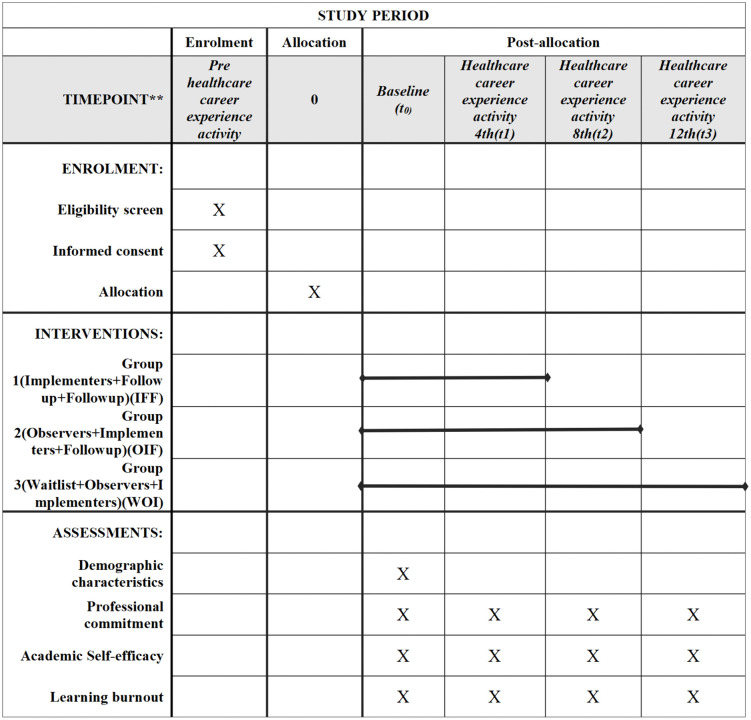
The schedule of enrollment, interventions, and assessments.

This study will utilize an open-label, randomized, stepped-wedge crossover design. Eligible nursing students will be randomly assigned in a 1:1:1 ratio to one of three groups: Group 1 (Implementers+Followup+Followup) (IFF), 2 (Observers+Implementers+Followup) (OIF) or 3 (Waitlist+Observers+Implementers) (WOI). In accordance with the stepped-wedge schedule, all groups will sequentially receive the intervention after transitioning through control conditions (Observer or Waitlist). The specific sequence of roles and intervention timing for each group is detailed in Fig 2. Outcomes will be measured at four time points: baseline (T0), and at three subsequent follow-up points (T1, T2, T3). The schedule of enrollment, interventions, and assessments is shown in Fig 3. This stepped-wedge design was chosen for two main reasons. First, it allows all participants to eventually receive the intervention, which is ethically preferable in educational studies. Second, it helps to control for time-related confounding variables. This protocol is reported in accordance with the recommendations for clinical trials guidelines (SPIRIT) [35].

### 3.2. Ethics

This study protocol was approved by the Medical Ethics Review Committee of Quanzhou Medical College (No. 2023003). Prior to enrollment, all participants will receive detailed information about the study and will be required to provide both verbal and written informed consent. Participants will be informed of their right to withdraw from the study at any time without any negative consequences. All data, including participant growth files will be stored securely and confidentially in an archives room, in accordance with the guidelines of the Medical Ethics Review Committee of Quanzhou Medical College. Access to the dataset is restricted to the principal researchers involved in this study to ensure confidentiality.

### 3.3. Participants

#### 3.3.1. Nursing students.

Participants are second-year nursing students from the school of nursing at a medical vocational college in China. The study aims to include all eligible students from this cohort who volunteer to participate. The inclusion criteria are as follows: (1) full-time nursing who have completed at least one academic year of study; (2) recruited through the national college entrance examination; and (3) provided written informed consent after understanding the study details. The exclusion criteria are: (1) students who drop out; (2) students who are suspended from school; or (3) students who change their major during the study period.

#### 3.3.2. Middle school students.

The experiencers for the activity will be recruited from a middle school near the medical vocational college. In collaboration with the school, the activity is held once every two weeks on weekends for students who volunteer to participate. To ensure a high-quality experience and due to venue limitations, each activity session is limited to a total of 18–36 experiencers. This group size is designed to be manageable and allows for the experiencers to be subsequently divided into three smaller teams for the different experiential modules.

### 3.4. Sample size

The sample size was estimated specifically for a GEE analysis using the longpower package in R statistical software (version 4.4.3). The calculation was designed to detect significant differences in the primary outcome among the three groups across four repeated measurement points. The parameters were set based on standard benchmarks: a significance level (α) of 0.05, a power of 0.80, and a standard medium effect size (δ = 0.50). Regarding the correlation structure, we adopted a conservative estimation. Given that professional commitment is a dynamic construct [36], we assumed a moderate within-subject correlation (ρ = 0.35) [37]. Based on these parameters, the minimum required sample size is 33 participants per group. To account for a potential 10% attrition rate, the final target sample size is set at 110 participants. This method of ensuring adequate statistical power aligns with recommendations in methodological literature [38,39].

### 3.5. Recruitment and allocation

#### 3.5.1. Nursing students.

Between June 2025 and June 2026, eligible students who agree to participate in the trial will be invited to join a QQ group as part of the volunteer pool. Researchers will create a random grouping table in advance. This table will include study ID numbers, names, and random numbers, along with group assignments. This list will be kept confidential, and access will be restricted to the research team. To ensure random assignment, the RAND function will be used to generate a random number for each individual’s ID. The eligible students will then be divided into three groups (Group 1, Group 2, or Group 3) using a 1:1:1 allocation method. Researchers will assign specific study ID numbers based on the order in which participants join the volunteer pool. They will be informed of their group assignments via QQ one week prior to the intervention. Additionally, students assigned to the implementer role will be further randomly allocated to one of three WeChat groups, each providing detailed implementation plans for different modules.

#### 3.5.2. Middle school students.

Upon reaching the recruitment target, the experiencers will be assigned to three equal-sized teams. Each team will begin at a different module and subsequently rotate through all three modules. The group assignments will be communicated to the head teachers, who will then notify the students.

### 3.6. Study procedure

#### 3.6.1. Development of the student-led healthcare career experience activity for middle school students.

**Development of the student-led healthcare career experience activity.** A preliminary framework for the healthcare career experience activity was established by the project leader and subsequently refined by a guidance team. The project team consisted of eight faculty members, all holding at least a master’s degree and possessing a minimum of five years of teaching experience in nursing education.

**Healthcare career experience activity content design.** For the content design, the Modified Brainstorming Technique was implemented [40]. An inventory of all available nursing positions and educational resources at the institution was compiled. The activities were specifically tailored to the cognitive and physical developmental stages of middle school students, referencing the *Curriculum Guidelines for Comprehensive Practical Activities in Primary and Secondary Schools* (Ministry of Education of the People’s Republic of China, 2017) [41]. Based on peer evaluations, the final content was structured into three experiential modules aligned with key life stages: Early Life, Life in Crisis, and Later Life.

**Healthcare career experience activity process design.** Kolb’s experiential learning theory was applied to create the student-led modules. The process for each station was structured to follow Kolb’s four stages: concrete experience, reflective observation, abstract conceptualization, and active experimentation. A comprehensive description of the activities is provided in Table 1.

**Table 1:** Comprehensive account of healthcare career experience activities.

Module	Station	Process
Early life experience	1. Childbirth pain & Labor Support	1. Concrete Experience (Observational): Watch the delivery process using VR glasses. 2. Reflective Observation: Share observations and initial feelings about the process. 3. Concrete Experience (Somatic): Experience simulated labor pain with a simulator. 4. Abstract Conceptualization: Summarize the standard nursing interventions for labor support, including breathing coaching and physical comfort measures. 5. Active Experimentation: Role-play as a nurse to provide labor support. Specifically, guide the fellow experiencer through Lamaze breathing rhythms during contractions and perform lower back massage to alleviate simulated pain.
	2. Neonatal care	1.Concrete Experience: Watch implementers demonstrate the correct procedure for bathing and handling a newborn model. 2.Reflective Observation: Share observations on the key safety steps and precautions taken by the implementer. 3.Abstract Conceptualization: Summarize the core principles of neonatal safety, focusing on head support, temperature control, and gentle handling. 4.Active Experimentation: Apply neonatal care principles by performing the standard newborn bathing sequence and executing the correct holding technique to support the head and neck.
Life in Crisis experience	1. CPR(cardio-pulmonary resuscitation)	1.Concrete Experience: Watch implementers demonstrate the full CPR process on a manikin. 2.Reflective Observation: Share observations on key technical actions like chest compression depth and rate. 3.Abstract Conceptualization: Summarize the critical principles of effective CPR, specifically the logic behind the “fast, deep, and consistent” compressions and the 30:2 ratio. 4.Active Experimentation: Execute high-quality chest compressions and ventilation on a manikin strictly adhering to the 30:2 cycle and standard depth requirements.
	2. Heimlich maneuver	1.Concrete Experience: Watch implementers demonstrate the Heimlich maneuver on an adult manikin. 2.Reflective Observation: Share observations on precise hand placement and the direction of force. 3.Abstract Conceptualization: Summarize the principle of creating an artificial cough to expel a foreign object from the airway. 4.Active Experimentation: Perform the abdominal thrust maneuver on the simulation manikin using correct hand positioning to dislodge the foreign body.
Later life experience	1. Geriatric Care & Mobility Support	1.Concrete Experience: Wear a senior-sensation simulator to experience the physical limitations of the elderly. 2.Reflective Observation: Share feelings about the sensory and mobility challenges experienced during the simulation. 3.Abstract Conceptualization: Summarize the nursing principles for fall prevention and mobility assistance for the elderly. 4.Active Experimentation: Act as a community nurse to assist the elderly. Perform a fall risk assessment on a fellow experiencer wearing the simulator and physically assist them to walk safely using a walker.
	2. Rehabilitation Guidance	1.Concrete Experience: Watch implementers demonstrate the use of rehabilitation training instruments. 2.Reflective Observation: Share observations on the specific purpose and target muscle groups of each instrument. 3.Abstract Conceptualization: Summarize the principles of rehabilitation nursing, focusing on promoting fine motor skills and maintaining joint mobility. 4.Active Experimentation: Role-play as a rehabilitation nurse. Instruct the fellow experiencer on the correct operation of the rehabilitation instruments to ensure effective fine motor skill training.

#### 3.6.2. Intervention.

**Fig 4:**
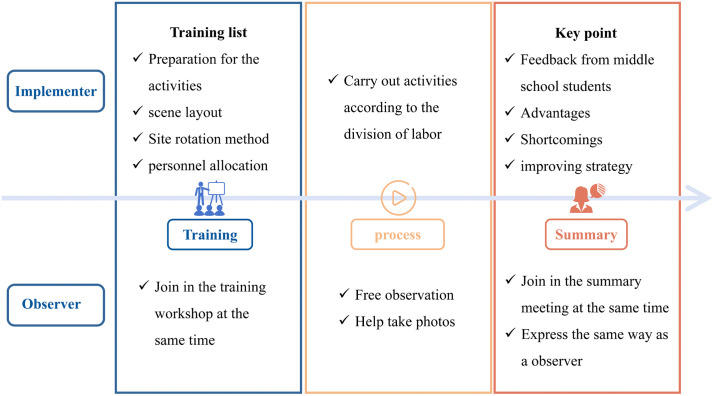
Roles of different groups and details in the healthcare career experience activity.

**Intervention Timing Arrangement.** The intervention timing will follow the stepped-wedge design detailed previously (Fig 2). Group 1 (IFF) will initially act as the intervention group (implementers), while Group 2 (OIF) and Group 3 (WOI) will begin in control conditions as Observers and Waitlist, respectively. Subsequently, Group 2 and Group 3 will cross over to the implementer role after the 4th and 8th activity sessions, respectively. After completing four activity sessions, nursing students have the option to continue their participation in a subsequent volunteer pool. The specific roles for each group at each phase are outlined in Fig 2 and Fig 4.

**Intervention group training.** A training manual for the healthcare career experience activities was developed. The manual includes four sections: activity planning, preparation, detailed implementation steps, and instructor capability assessment. The nursing students will be required to independently study the first two sections using the manual and associated video resources via the Super Star Learning App. The third section, detailing the specific implementation steps for each module, will be taught by the research team members designated for each module. Following this training, all nursing students must pass a practical assessment that simulates the career experience activities before they can act as implementers.

**Intervention implementation.** Each experience module will be led by a designated module leader responsible for organization and quality oversight. At each station, three implementers will be required. One will demonstrate the procedures and explain the materials, while the other two will assist the experiencers with equipment and clarify content. Each implementer will also be responsible for facilitating the reflective stages of the learning cycle with one to two experiencers. To ensure equal opportunities, the roles of the three implementers will be rotated when the next group of experiencers arrives. The experiencers will engage in the activities according to the prescribed segments, as detailed in Table 2. Each station accommodates 3–6 participants per session, with each session lasting approximately 20 minutes in total.

**Table 2:** The roles of implementers and experiencers in the implementation process.

Step	Module	Content	Location	Time (min)	Action of implementer （nursing students）	Action of experiencer (middle school students)
1	Opening	The aim of activities and announcements	Lecture hall	15	Module leader: Introduction	Assigned to three teams
2	Early life experience	Brief introduction	Medical virtual simulation training center	5	Module leader: Introduction	Listening
2A		Childbirth pain & Labor Support		20	A: Demonstrates the delivery process and labor support skills. B and C: Assist experiencers with equipment and facilitate reflection.	Watch (VR experience) Share (Reflections) Summarize (Support principles) Guide fellow experiencer (Lamaze breathing & Massage)
2B		Neonatal care		20	A: Demonstrates bathing and handling newborns. B and C: The same as the above.	Watch demonstration Share (Reflections) Summarize (Safety principles) Perform bathing and holding
3	Life in Crisis experience	Brief introduction	Emergency training center	5	Module leader: Introduction	Listening
3A		CPR(cardio-pulmonary resuscitation)		20	A: Demonstrates the full CPR process B and C: the same as the above.	Watch demonstration Share (Reflections) Summarize (CPR principles) Perform compressions & ventilation
3B		Heimlich maneuver		20	A: Demonstrates the Heimlich maneuver. B and C: The same as the above.	Watch demonstration Share (Reflections) Summarize (Principles) Practice the Heimlich maneuver
4	Later life experience	Brief introduction	Elderly care training center	5	Module leader: Introduction	Listening
4A		Geriatric Care & Mobility Support		20	A: Demonstrates fall prevention and mobility assistance. B and C: the same as the above.	Watch (Wear simulator) Share (Reflections) Summarize (Nursing principles) Assist fellow experiencer (Walking)
4B		Rehabilitation Guidance		20	A: Demonstrates rehabilitation training instruments. B and C: the same as the above.	Watch demonstration Share (Reflections) Summarize (Rehab principles) Instruct fellow experiencer on using instruments
5	Summary	Sharing the moments during the activities	Lecture hall	15	Module leader: Summary	Watch the video

### 3.7. Data collection and management

Prior to the study, designated researchers will receive standardized training in data collection procedures. Each nursing student will maintain a paper-based growth file, which is anonymized using a unique study ID number. This file contains the self-reported questionnaires collected at each time point, as well as qualitative materials such as feedback from middle school students, academic awards, post-intervention reflections, and a letter written to their future self. These qualitative materials will be used to triangulate and provide context to the quantitative data, partially mitigating potential self-report biases.

Following each data collection point, researchers will check the data for completeness. The nursing students will be asked to supply any missing information immediately to prevent data loss. To ensure confidentiality, all completed growth files will be stored in a locked, secure research data room, with access restricted to the principal researchers. Outcomes will be assessed at baseline (T0) and three subsequent time points (T1, T2, and T3), which correspond to the completion of the 4th, 8th, and 12th activity sessions respectively, as detailed in Table 3.

**Table 3:** Timeline of measurement details.

Timeline	Group	Status	Demographic characteristics	Professional commitment	Academic Self-efficacy	Learning burnout
T0	Group 1	Implementers	√	√	√	√
	Group 2	Observers	√	√	√	√
	Group 3	Waitlist	√	√	√	√
T1	Group 1	Implementers	–	√	√	√
	Group 2	Observers	–	√	√	√
	Group 3	Waitlist	–	√	√	√
T2	Group 2	Implementers	–	√	√	√
	Group 3	Observers	–	√	√	√
	Group 1	Followup	–	√	√	√
T3	Group 3	Implementers	–	√	√	√
	Group 1	Followup	–	√	√	√
	Group 2	Followup	–	√	√	√

### 3.8. Outcomes and measures

#### 3.8.1. Main outcome: professional commitment.

The primary outcome measure in this study is the change in professional commitment among nursing students. This outcome will be measured using the Undergraduate Professional Commitment Scale, developed by Lian R et al [42]. The scale is organized into four dimensions: emotional commitment, continuing commitment, ideal commitment, and normative commitment. It comprises 27 items, with responses rated on a 5-point Likert scale ranging from 1 (“completely inconsistent”) to 5 (“completely consistent”). Higher scores indicate greater professional commitment. The scale demonstrates strong reliability, with a Cronbach’s α coefficient of 0.927.

#### 3.8.2. Secondary outcome.

**Academic self-efficacy** This outcome will be measured using the Academic Self-efficacy Questionnaire compiled by Liang YS and Zhou ZK [43]. This questionnaire was developed with reference to the academic self-efficacy scale created by Pintrich and DeGroot (1990). It consists of 22 items divided into two dimensions: self-efficacy of learning ability and self-efficacy of learning behavior. The questionnaire has a Cronbach’s α coefficient of 0.89, indicating strong reliability. Responses are collected using a 5-point Likert scale, ranging from 1 (“completely disagree”) to 5 (“strongly agree”), with higher scores reflecting a greater sense of self-efficacy.

**Learning burnout** This outcome will be assessed using the Learning Burnout Scale for college students, developed by Lian Rong in 2005 [44]. This scale includes three dimensions: depression (8 items), improper behavior (6 items), and low sense of achievement (6 items), totaling 20 items. A 5-point Likert scale is used, with responses ranging from 1 (“completely inconsistent”) to 5 (“fully consistent”). The total score ranges from 20 to 100, with higher scores indicating a greater degree of learning burnout. The scale demonstrates strong reliability, with a total Cronbach’s α coefficient of 0.865. The three extracted common factors explain 51.45% of the total variance. In this study, the total Cronbach’s α coefficient for the scale is 0.868.

### 3.9. Statistical analysis

All statistical analyses will be performed using SPSS version 27.0. Demographic variables will be summarized using descriptive statistics. In accordance with the Intention-to-Treat (ITT) principle, all randomized participants will be included in the analysis. Missing data will be handled using the GEE model under the Missing at Random (MAR) assumption. The GEE model will be used to compare differences between and within groups, controlling for time effects and baseline scores of the outcome variables (professional commitment, academic self-efficacy, and learning burnout). To examine the specific impact of each intervention role, the Group variable will be analyzed as a three-level categorical factor (Implementer, Observer, Waitlist). The interaction effect between time and group allocation will also be examined. Post-hoc multiple comparisons between groups will be conducted using the Bonferroni correction. For all analyses, statistical significance is set at a p-value of < 0.05.

### 3.10. Data sharing and dissemination

The reporting of the trial results will adhere to the consolidated standard of reporting trials (CONSORT) guidelines. Following the completion of data analysis, the findings are intended for publication in peer-reviewed journals and for presentation at academic conferences. The datasets generated from this study will be made available by the corresponding author upon reasonable request.

## 4. Discussion

This study protocol introduces a student-led healthcare career experience activity. The design integrates Life Cycle Theory for its structure and Kolb’s Experiential Learning Theory for its process. This PAL model aims to offer a sustainable solution to resource scarcity. It also helps student implementers bridge the gap between theory and practice.

A key strength of this study is its intervention design, grounded in Scaffolding Theory [45] and Social Cognitive Theory [46]. This framework is designed to support program sustainability while allowing for multi-dosage assessment. The proposed student-led model directly addresses the issue of resource scarcity in career experience activities [17,19]. Traditional programs depend heavily on continuous faculty input. In contrast, this model seeks to establish a self-sustaining cycle. Through the integration of vicarious experiences into the peer-assisted framework, the Observer phase is positioned as a necessary preparatory cognitive scaffold. In this mechanism, current implementers are expected to not only guide middle school participants but also implicitly train the observers. This process is anticipated to foster a system of autonomous operation, which has the potential to reduce the long-term demand on faculty and enhance scalability. Furthermore, treating the Observer as an active comparator allows for the examination of how different intervention dosages in an open-label setting might impact outcomes such as professional commitment.

Furthermore, the student-led approach transforms nursing students from passive learners into active practitioners. This is critical in a practice-oriented profession like nursing. Early practical application is essential for a smooth transition from student to professional [47–49]. Research also indicates that early exposure to the professional practice environment fosters a sense of belonging and confidence [50]. By providing an early practice scenario during the theoretical learning stage, this study serves to reinforce the nursing students’ future roles as nurses. Through repeated engagement in this practice, they can internalize their knowledge, thereby deepening their identification with the nursing profession and enhancing their professional commitment [51,52].

The methodological rigor in developing the intervention is another notable strength. The study applied Life Cycle Theory to systematically compile a list of nursing roles. This helps nursing students develop a more holistic understanding of the profession. In addition, a Modified Brainstorming Technique was used for the activity design. This structured approach ensured the scientific rationale of the intervention content. This development process offers a valuable reference for future researchers. By focusing on changes in professional commitment during this practice, the study also allows for a deeper understanding of Experiential Learning Theory.

Finally, the protocol incorporates several specific measures to control for potential biases, particularly those arising from the open-label design. The choice of a stepped-wedge, waitlist control design is itself a key bias-control strategy. This design ensures that all nursing students eventually receive the same intervention dosage, helping to reduce dose-response bias. It also helps minimize a uniform Hawthorne effect, as groups are in different roles at any given time. To further mitigate researcher and Hawthorne effects, the role of the research team members is strictly defined. They act only as coordinators and do not directly participate in the intervention activities, thus minimizing their influence on the nursing students. Furthermore, the protocol includes a direct check for the Hawthorne effect, the nursing students are asked in their growth files whether awareness of being studied affected their participation. To address outcome expectation bias, data is triangulated by comparing the quantitative results from questionnaires with qualitative insights from the nursing students’ reflections and feedback records. This mixed-methods approach enhances the overall credibility and reliability of the findings.

## 5. Limitations

Several limitations of this study protocol should be acknowledged. First, the follow-up period is short. Long-term outcomes, such as career retention after graduation, are not measured. Second, the outcomes rely exclusively on self-reported questionnaires, which may introduce social desirability or recall bias. Third, the study is conducted at a single college. This may limit the generalizability of the findings to other settings. Fourth, the trial is open-label, so blinding is not possible, and the potential for baseline imbalances remains. Finally, the use of a waitlist control group poses a risk. Participants might withdraw or seek other opportunities, which could lead to attrition or self-selection bias.

## 6. Implications

For practice, this student-led activity offers a feasible and low-cost model for the early cultivation of professional commitment. Nursing educators could integrate this activity into career planning courses. This would help nursing students better understand their future professional roles and reduce misconceptions. The model also fosters strong academic-practice partnerships. For future research, this study highlights several directions. Future work should use a longitudinal design to track long-term outcomes like career retention. Researchers could also incorporate more objective measures, such as academic performance records or feedback from supervisors. Finally, conducting multi-center trials is crucial to enhance the generalizability of the findings.

## 7. Conclusion

This study protocol details a structured, student-led healthcare career experience activity designed to enhance professional commitment among nursing students. The trial evaluates a peer-assisted learning model. It is expected to provide empirical evidence for a feasible and sustainable approach to career education. This approach also simultaneously addresses resource limitations. Ultimately, this research aims to inform the development of effective early career interventions to help stabilize the future nursing workforce.

## Supporting information

S1 File SPIRIT-Checklist. (DOC)
